# Cardiovascular Complications in ADPKD

**DOI:** 10.1016/j.ekir.2025.06.054

**Published:** 2025-07-05

**Authors:** Niloufar Ebrahimi, Yasar Caliskan, Pranav S. Garimella, Sol Carriazo, Fouad T. Chebib, Giv Heidari Bateni, Neera K. Dahl, Anjay Rastogi, Amir Abdipour, Sayna Norouzi

**Affiliations:** 1 Department of Medicine, Division of Nephrology, Loma Linda University Medical Center, Loma Linda, California, USA; 2 Saint Louis University Center for Abdominal Transplantation, Saint Louis University, St. Louis, Missouri, USA; 3 Division of Nephrology and Hypertension, Department of Medicine, University of California San Diego, San Diego, California, USA; 4 Division of Nephrology, University Health Network, Toronto, Ontario, Canada; 5 Division of Nephrology and Hypertension, Mayo Clinic, Jacksonville, Florida, USA; 6 Division of Cardiology, Arrowhead Regional Medical Center, California University of Science and Medicine, California, USA; 7 Division of Nephrology and Hypertension, Mayo Clinic, Rochester, Minnesota, USA; 8 Department of Medicine, University of California Los Angeles, Los Angeles, California, USA

**Keywords:** autosomal dominant polycystic kidney disease, cardiovascular complications, hypertension, left ventricular hypertrophy, valvular abnormalities

## Abstract

Autosomal dominant polycystic kidney disease (ADPKD), primarily caused by mutations in the *PKD1* or *PKD2* gene, is among the most common hereditary kidney diseases worldwide and is associated with significant extrarenal manifestations, including cardiovascular disease. In patients with ADPKD, cardiovascular disease is the major cause of mortality and is associated with a high burden of comorbidities. Cardiovascular manifestations include hypertension, which may lead to left ventricular hypertrophy (LVH) and diastolic dysfunction, as well as valvular abnormalities, aortic aneurysm, and pericardial effusion (PCE). The cardiovascular manifestations can present early as part of the ADPKD manifestation or later because of chronic kidney disease (CKD) progression. Early detection of cardiovascular manifestations can play a pivotal role in better management of these patients. Hypertension in ADPKD might start at an early age and is driven by a complex interplay of polycystin (PC) dysfunction, intracellular signaling disruptions, and activation of the renin-angiotensin-aldosterone system (RAAS), which can contribute to vascular structural changes and impaired endothelial function. Valvular involvement exhibits a bimodal pattern of age distribution, with early manifestations occurring in younger patients likely linked to genetic factors and later complications emerging as part of CKD progression. This review explores cardiovascular complications in ADPKD, emphasizing the need for early detection, and briefly provides an overview of tailored management approaches to improve outcomes in this high-risk population.

ADPKD is the most common monogenic kidney disease and an important cause of end-stage kidney disease, caused by mutations in the *PKD1* or *PKD*2 genes in the majority of the cases, leading to dysfunction of their encoded proteins, PC-1 and PC-2. Other minor genes such as *ALG5, ALG9*, *DNAJB11, GANAB, IFT140,* and *NEK8* account for the new polycystic kidney disease nomenclature and approximately 7% of the cases.^1^, ^2^, ^3^ Lack of functional PCs results in the formation of numerous bilateral kidney cysts, leading to kidney enlargement, and kidney function decline.^1^ Tolvaptan, an arginine vasopressin hormone V2 receptor blocker, is currently the only disease-modifier treatment approved for ADPKD with a high risk of progression.^4^ In addition, the disease is closely associated with significant extrarenal manifestations, including liver cysts and polycystic liver disease, intracranial aneurysms (ICAs), and cardiovascular dysfunction, among others.^1^ Loss of PC-1 or PC-2 proteins also contributes to developing extrarenal manifestations in ADPKD.^5^ Notably, a reduction in PC-1 is associated with the early onset of cardiovascular disease.^5^

Cardiovascular complications are the leading cause of morbidity and mortality in this patient population, making them an essential focus in ADPKD management.^5^ These complications include the early onset of hypertension, which can later lead to LVH and diastolic dysfunction, valvular abnormalities, aortic root dilatation, and dilated cardiomyopathy.^6^^,^^7^ Patients with APDKD also have an elevated risk for arterial aneurysms and dissections. Notably, arterial aneurysms, especially ICAs, can occur independently of any valvular abnormalities. In addition, PCE can be observed in individuals with ADPKD.^6^ This elevated cardiovascular risk should urge physicians to prioritize early screening and timely management to improve patient’s outcomes.^8^ In Figure 1, we show the potential cardiovascular complications associated with ADPKD.

**Figure 1:**
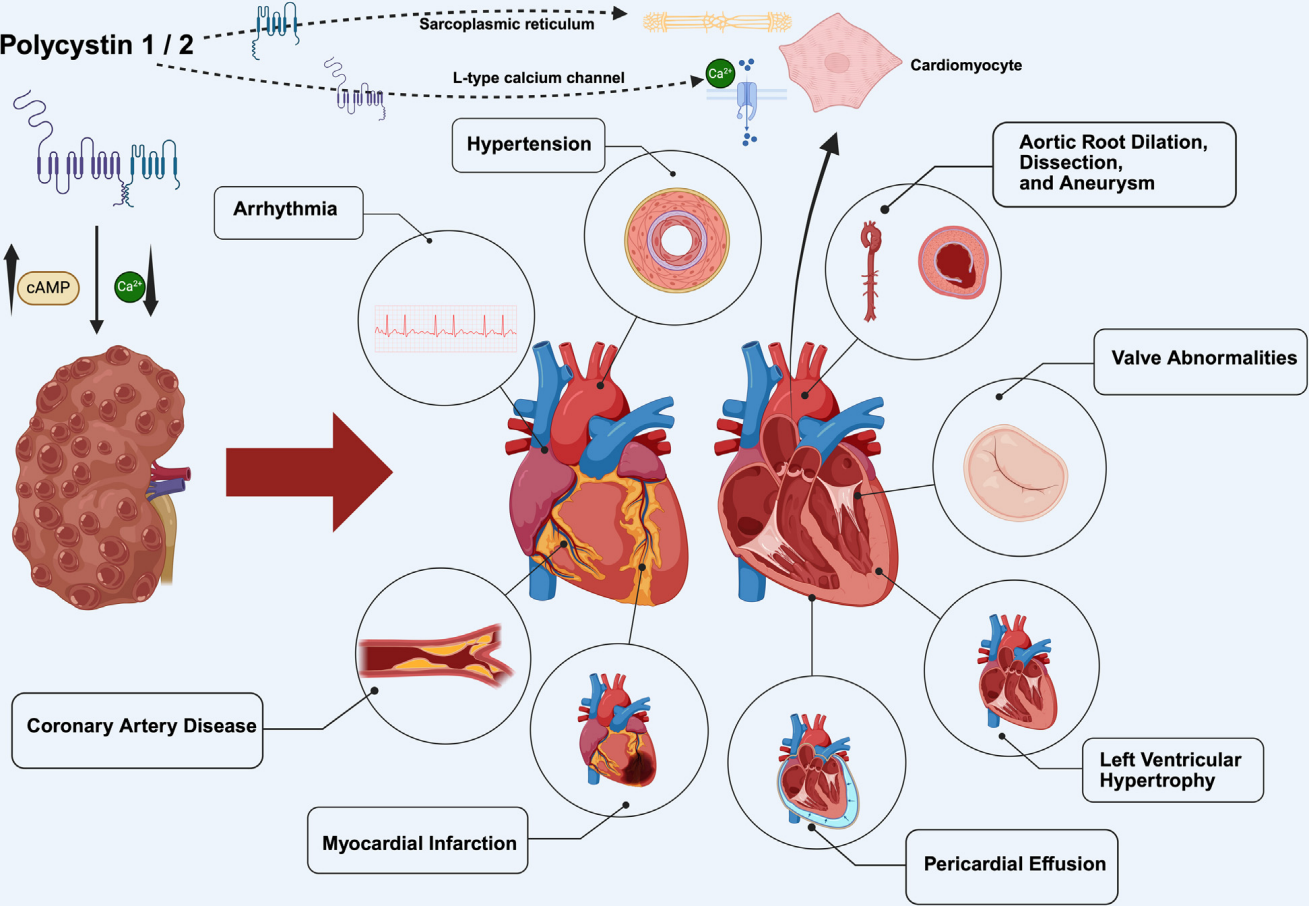
Cardiovascular complications in ADPKD arise from *PKD1* and *PKD2* mutations. PC-1 stabilizes L-type calcium channels in cardiomyocytes; its dysfunction reduces the α1C subunit, promoting ventricular hypertrophy and fibrosis. PC-2, localized to the sarcoplasmic reticulum, modulates cardiac contractility and synchronization, with its loss resulting in reduced shortening and desynchronization. These polycystin alterations may drive cardiac remodeling in ADPKD independent of hypertension or kidney function impairment. ADPKD, autosomal dominant polycystic disease.

### Hypertension

Hypertension can occur early in ADPKD with preserved glomerular filtration rate (GFR) and is closely linked to kidney function decline, as well as to an increased risk of cardiovascular mortality.^9^ Having hypertension before the age of 35 years is a risk factor for more rapid progression, as defined in the predicting renal outcome in polycystic kidney disease score.^9^ In addition, different cohorts have reported an association between hypertension and greater kidney volumes, rates of kidney growth, and progression to kidney failure, compared with patients with ADPKD with normal blood pressure (BP).^10^

Dysfunctional PC-1 and PC-2, transmembrane glycoproteins located in the primary cilium of kidney tubular epithelial cells, lead to a disruption in the intracellular and ciliary signaling pathways by lowering intracellular calcium levels and increasing cyclic adenosine monophosphate, which promotes abnormal cell proliferation and fluid secretion into cysts.^11^^,^^12^ The abnormal function of PC-1 and PC-2 in blood vessels results in altered vascular structure and function, reduced nitric oxide levels, and impaired endothelial response.^13^ Moreover, kidney cyst growth triggers nephron obstruction and microvascular ischemia, stimulating the activation of the RAAS, further leading to hypertension in patients with ADPKD.^13^

In the HALT-PKD study A, a randomized, double-blinded clinical trial with a 2 by 2 factorial design, researchers evaluated the efficacy and safety of combined treatment with a dual RAAS inhibition angiotensin-converting enzyme inhibitor (ACEI) with an angiotensin receptor blocker (ARB) versus ACEI monotherapy. A total of 558 hypertensive patients with ADPKD between the ages of 15 to 49 years, with an estimated GFR (eGFR) > 60 ml/min per 1.73 m^2^ were enrolled and randomized in the study. The study also compared the effect of standard (120/70 to 130/80 mm Hg) versus intensive (95/60 to 110/75 mm Hg) BP targets on total kidney volume (TKV) in patients with ADPKD.^14^ Both treatment groups had similar rates of TKV increase (6.0% vs. 6.2% /yr, *P* = 0.52) and decline in eGFR (−3.00 vs. −2.86 ml/min per 1.73 m^2^/yr, *P* = 0.55).^14^ However, the low BP group demonstrated significant benefits, including a slower annual increase in TKV (5.6% vs. 6.6%, *P* = 0.006) and a greater reduction in urinary albumin excretion (UAE) rate (−3.77% vs. 2.43% /yr, *P* < 0.001) compared with the standard BP group. In addition, the intensive BP control group maintained differences of 13.4 mm Hg in systolic and 9.3 mm Hg in diastolic pressure at 96 months.^14^ Intensive BP control was reported to be tolerable and safe, showing similar side-effects to the standard BP group. However, the incidence of dizziness and light-headedness from the study results was notably higher in the intensive BP control group than in the standard BP group (80.7% vs. 69.4%, *P* = 0.002).^14^

Long-term follow-up of participants in the HALT-PKD trial, 10 years after the completion of the trial, failed to demonstrate that either dual RAAS inhibition or intensive lowering BP reduced risk of kidney failure or mortality.^15^ However, BP management in the groups was not monitored after study completion.^16^ Based on the results of the HALT-PKD trials, the target BP goal for patients with ADPKD, who are aged 18 to 49 years with preserved kidney function (eGFR ≥ 60 ml/min per 1.73 m^2^) is < 110/75 mm Hg.^17^ In other patients with ADPKD (aged ≥ 50 years, or those with stage 3 CKD or higher) a target BP ≤ 120/80 mm Hg is recommended, in accordance with the Kidney Disease: Improving Global Outcomes (KDIGO) guidelines for CKD (grade 2C).^17^

RAAS inhibitors, associated with nephroprotection in diabetic and nondiabetic kidney disease, are the cornerstone in BP management among patients with ADPKD.^18^ A systematic review of randomized controlled trials evaluating antihypertensive treatments in ADPKD included 10 trials with 1386 participants selected after reviewing 45 full-text articles and 197 records. The studies had a mean follow-up of approximately 4 years (range: 0.5–8 years) and featured balanced male-to-female ratios. No significant differences were observed in eGFR among the antihypertensive treatments, with eGFR changes of −8.00 (95% confidence interval [CI]: −18.05 to 2.05) ml/min per 1.73 m^2^ for ACEI versus placebo and −5.39 (95% CI: −25.96 to 15.17) ml/min per 1.73 m^2^ for ACEI versus β-blocker. However, ARBs and ACEIs significantly reduced UAE compared with calcium channel blockers, with ARBs showing a mean decrease of −238.00 (95% CI: −394.61 to −81.39) mg/24 h and ACEIs showing −134.00 (95% CI: −176.01 to −91.99) mg/24 h compared with calcium channel blockers. ACEIs showed a more significant reduction in left ventricular mass index (LVMI) with a mean difference of −27.00 g/m^2^ (95% CI: −43.07 to −10.93) compared with calcium channel blockers. Rigorous BP control (target < 120/80 mm Hg) resulted in a greater decrease in LVMI compared with standard control (mean difference: −14.56, 95% CI: −27.06 to −2.06), whereas eGFR and UAE were similar between the groups. Bayesian probability analysis ranked ARBs as the most effective treatment for eGFR, UAE, and systolic BP.^18^

These findings suggest that though intensive BP control and RAAS blockade may not significantly alter short-term eGFR trajectories, they offer cardiovascular benefits and reductions in proteinuria that are clinically relevant in ADPKD management. Choosing antihypertensive agents that consider kidney outcomes and cardiovascular protection is crucial, particularly in patients with early-stage disease. A more nuanced understanding of class-specific effects, primarily on surrogate markers such as UAE and LVMI, highlights this population's need for individualized BP management strategies.

According to the KDIGO 2025 ADPKD guidelines, standardized office BP measurements are preferred over routine assessments (grade 1B). For patients with ADPKD aged 18 to 49 years with CKD stages G1–G2 and elevated BP (> 130/85 mm Hg), a target BP ≤ 110/75 mm Hg, measured by home BP monitoring, is recommended if tolerated (grade 1D). For patients aged ≥ 50 years with CKD stages G1–G5, a mean systolic BP target < 120 mm Hg based on standardized office measurements is advised (grade 2C). RAAS inhibitors (ACEI or ARBs) are recommended as first-line agents to achieve these BP targets (grade 1C).^3^

Nonpharmacological measures such as low dietary sodium intake, dietary approach to stop hypertension diet, regular physical activity, maintaining a normal weight, smoking cessation, and stress management are strongly recommended for BP control in the general population.^19^ In the HALT-PKD study A, sodium intake was quantified through annual 24-hour urine collections, which enabled centralized measurements of urinary sodium excretion.^14^ Lower sodium intake (< 2 g/d), as observed among participants, was associated with more favorable kidney disease outcomes; however, it is important to note that sodium restriction was not a randomized intervention in this study.^14^

Reducing salt intake has been shown to lower BP and enhance the renoprotective effects of RAAS blockade, thereby contributing to improved clinical outcomes in CKD management. An observational cohort study of 589 patients with ADPKD, including 288 participants from the Developing Interventions to Halt Progression of ADPKD 1 trial, assessed the relationship between salt intake and kidney function decline. Over a median 4-year follow-up, salt intake was significantly associated with an annual decline in eGFR of −0.11 ml/min per 1.73 m^2^/g of salt consumed (95% CI: −0.20 to −0.02). These findings suggest that higher salt intake is linked to accelerated kidney function decline in patients with ADPKD.^20^

The KDIGO 2025 ADPKD guidelines recommend limiting dietary sodium intake to < 2.3 g/d (equivalent to ≤ 5 g of salt daily) to support optimal BP control and kidney health.^3^

According to a nationwide, multicenter, retrospective observational study conducted in Japan, patients with ADPKD who progressed to CKD-G5 were divided into 3 groups: the tolvaptan-continued group, the tolvaptan-discontinued group, and the non-tolvaptan group. The results demonstrated that the eGFR slope remained stable before and during the progression to CKD-G5 in the non-tolvaptan group. In the tolvaptan-discontinued group, the eGFR slope accelerated from −3.3 (−4.3 to −2.5) ml/min per 1.73 m^2^/yr before CKD-G5 to −5.3 (−6.7 to −3.8) ml/min per 1.73 m^2^/yr during CKD-G5. In contrast, the eGFR slope in the tolvaptan-continued group showed no significant change, remaining at −3.9 (−5.4 to −2.6) ml/min per 1.73 m^2^/yr before CKD-G5 and −3.8 (−5.4 to −3.4) ml/min per 1.73 m^2^/yr during CKD-G5. These findings suggest that the continuation of low-dose tolvaptan may effectively help suppress the deterioration of kidney function in patients with ADPKD with CKD-G5.^21^

To slow CKD progression in ADPKD, current KDIGO 2025 ADPKD guidelines recommend using tolvaptan in adults with an eGFR ≥ 25 ml/min per 1.73 m^2^ who are at risk for rapid progression. In addition, in patients with eGFR ≥ 30 ml/min per 1.73 m^2^ and no contraindications, an increase in daily water intake to 2 to 3 L may help suppress endogenous vasopressin, potentially exerting a protective effect on kidney function. The guidelines also advise against the use of certain therapies that have not demonstrated benefit in slowing ADPKD progression or may carry unnecessary risks in this population, including mammalian target of rapamycin inhibitors, statins (when not otherwise indicated), somatostatin analogs, and metformin in non-diabetic individuals. These recommendations highlight the importance of targeted interventions and the avoidance of ineffective or potentially harmful therapies in the management of ADPKD-related CKD.^3^

### LVH and Remodeling

LVH, typically related with hypertension, and other comorbidities such as cardiac valvular disease, is characterized by myocyte hypertrophy and perivascular and interstitial fibrosis and has been reported to be associated with elevated morbidity and cardiovascular mortality.^22^ The prevalence of LVH in ADPKD has been reported to have notable variability among different studies, up to 66%.^22^ LVH, particularly LVMI, has been associated with mortality in hypertensive patients.^8^^,^^23^

In cardiomyocytes, PC-1 stabilizes and regulates the degradation of L-type calcium channels. Mutations leading to PC-1 dysfunction reduce the α1C subunit of the L-type calcium channel in these cells, contributing to the progression of ventricular hypertrophy and fibrosis.^24^ PC-2, located to the sarcoplasmic reticulum, and its loss leads to reduce cardiac shortening and cardiac desynchrony.^8^^,^^25^^,^^26^ Therefore, alterations in PC levels within the heart may directly contribute to cardiac remodeling in patients with ADPKD without hypertension or kidney failure. However, early-onset hypertension, which is strongly associated with LVH, plays a key role in the progression of LVH, and further LV diastolic dysfunction.^27^^,^^28^

Although LVH secondary to hypertension is a recognized cardiovascular manifestation in ADPKD, emerging evidence suggests that the condition also involves broader cardiac remodeling processes.^13^ Cardiac remodeling encompasses hypertrophic changes in myocardial mass and alterations in myocardial structure, fibrosis, and function. Several molecular mediators implicated in ADPKD may contribute to these remodeling processes.^13^ Elevated fibroblast growth factor 23 has been associated with accelerated cyst growth and adverse kidney function outcomes.^29^ In addition, it promotes LVH by activating the calcineurin–nuclear factor of the activated T-cell pathway signaling pathway, suggesting a direct role in structural myocardial remodeling.^29^ Elevated fibroblast growth factor 23 levels are also linked to increased arterial stiffness and atherosclerosis, independent of soluble Klotho levels.^30^ Fibroblast growth factor 23 can suppress parathyroid hormone secretion by acting on the parathyroid gland, which expresses both Klotho and fibroblast growth factor receptors. This interaction involves the activation of the MAPK pathway and the upregulation of calcium-sensing and vitamin D receptors, leading to decreased parathyroid hormone synthesis and parathyroid cell proliferation. However, in CKD, including ADPKD, this regulatory mechanism may be disrupted, contributing to secondary hyperparathyroidism.^31^ The interplay between these factors contributes to disturbances in mineral metabolism and cardiovascular complications, including vascular calcifications observed in ADPKD.^32^

In addition, genetic polymorphisms in the *ACE* gene, particularly the homozygous D allele, are linked with elevated ACE levels and may predispose to LVH and maladaptive myocardial changes in ADPKD.^33^ Galectin-3, another key mediator, is expressed in the kidney cystic epithelium and is associated with primary cilia dysfunction.^13^^,^^34^ Beyond its roles in the kidney, galectin-3 contributes to myocardial remodeling by promoting inflammation, fibrosis (via TGF-β signaling), and collagen production.^13^^,^^34^ Elevated circulating levels of galectin-3 are linked to CKD progression and myocardial dysfunction, and preclinical data suggest that galectin-3 inhibition may ameliorate cardiac abnormalities in ADPKD.^13^^,^^34^ These findings indicate that the cardiovascular involvement in ADPKD extends beyond concentric hypertrophy, including complex remodeling processes that warrant further investigation.^13^

The HALT-PKD study A reported a 3.9% prevalence of LVH. The study showed that the low BP group experienced a more significant LVMI reduction than the standard BP group (−1.17 vs. −0.57 g/m^2^/yr, *P* < 0.001). Therefore, intensive BP control was associated with a larger reduction in LVMI than standard control.^14^

Another study reported the prevalence of LVH in 126 patients with ADPKD, using echocardiography and evaluated the relationship with ADPKD severity.^35^ The median age and eGFR were 46 years and 63 ml/min per 1.73 m^2^, respectively; 78% of the study participants had hypertension with the median systolic BP of 125 mm Hg (interquartile range: 116–133). LVH was detected in 21.4% of participants, without significant differences between those with and without hypertension (*P* = 0.8), and the rate remained the same (21.4%) after excluding 21 individuals with known cardiac disease. Greater TKV and lower eGFR were significantly correlated with increased LVMI (*P* = 0.016 and *P* < 0.001, respectively). In multiple linear regression models adjusting for potential confounders, including BP, larger TKV was independently associated with higher LVM (β = 0.19, *P* = 0.04).^35^

In a cohort of 141 patients with ADPKD, 66% were found to have LVH, as indicated by an interventricular septal wall thickness at end diastole (IVSd) > 10 mm. This was significantly higher than 55% in the control group. The study found that IVSd was significantly greater in men than in women in both groups, and was correlated with disease severity, as patients in higher Mayo classes (MIC 1C–1E) had larger IVSd values. In addition, IVSd negatively correlated with eGFR, indicating that patients with more advanced CKD stages had more significant hypertrophy. Age also contributed to an increase in IVSd in both patients with ADPKD and controls. In multivariate analysis, whereas TKV and sex remained significant predictors of LVH, age and eGFR were not.^28^ The main differences between the prevalence reported across studies, appear to be primarily related to the imaging modalities used for LVH diagnosis (magnetic resonance imaging in HALT-PKD and echocardiography in other studies), as well as variations in the use of RAAS inhibitors.^22^

### Valve Abnormalities

Valvular abnormalities are common in both children and adults with ADPKD.^36^ In patients with APDKD, valvular heart disease shows a bimodal age distribution characterized by early onset and a late course.^8^^,^^37^^,^^38^ Valvular disease in the early stages is likely attributable to underlying gene mutations and encompasses valvular prolapse (usually mitral valve), often progressing to valvular regurgitation.^37^ A recent study on kidney transplant recipients reported that patients with ADPKD exhibited the highest prevalence of mitral valve prolapse (MVP) at 5.8%, highlighting a significant burden of MVP in this population compared with a nontransplant ADPKD group of patients.^5^ Posttransplant data also suggest a higher incidence of mitral valve involvement in ADPKD compared with other cardiac etiologies of kidney failure, underscoring the potential role of genetic defects.^5^

Notably, MVP has previously been reported in 20% to 30% of individuals with ADPKD. However, more recent studies applying updated diagnostic criteria for MVP have demonstrated significantly lower prevalence rates, with 1% observed in pediatric cohorts and 3.4% in adults, aligning closely with the general population.^3^^,^^37^

Valvular involvement is also observed in the later stage of the disease. This includes degenerative mitral and aortic valve disease, and it is usually secondary to the progression of CKD and kidney failure.^37^

The mechanism underlying valvular abnormalities in patients with ADPKD involves myxomatous degeneration characterized by the loss and disruption of collagen in the histological examination of mitral and aortic valve tissues. This degeneration is likely related to the expression of PC-1 and PC-2 proteins.^39^^,^^40^ Both proteins are found in cardiac myocytes and valvular myofibroblasts, suggesting that a genetic defect affecting these proteins may contribute to the observed myxomatous degeneration of mitral valve tissue in patients with ADPKD. This degeneration leads to structural alterations in the valve, potentially resulting in functional abnormalities such as MVP.^39^^,^^40^ The development of MVP in patients with ADPKD, particularly in those with normal BP and younger children, is believed to reflect the role of PCs in cardiac development rather than underlying structural defects or valvular complications associated with hypertension.^38^^,^^41^ Moreover, genes involved in primary ciliogenesis have been implicated in MVP beyond ADPKD contexts. Notably, the cilia gene, *DZIP1* has been identified as a causative gene in idiopathic MVP. This finding supports the hypothesis that defects in primary cilia contribute to mitral valve involvement in ADPKD.^37^

Pfeferman *et al.*^36^ assessed echocardiographic parameters in a large cohort of normotensive and hypertensive patients with ADPKD and explored their associations with clinical and laboratory data. A total of 294 young patients with ADPKD (120 males, 174 females, average age: 41.0 ± 13.8 years) were included, with 67.6% being hypertensive and having significantly lower eGFR compared with normotensive patients. MVP was identified in 3.4% of patients, whereas mitral, aortic, and tricuspid valve regurgitation occurred in 15.3%, 4.8%, and 16.0% of patients, respectively. There were no statistically significant differences between hypertensive and normotensive groups in the prevalence of mitral regurgitation (17.6% vs. 10.5%, *P* = 0.08) or tricuspid regurgitation (16.6% vs. 14.7%, *P* = 0.41). However, aortic regurgitation was significantly more common in hypertensive patients (6.5% vs. 1.0%, *P* = 0.03), whereas MVP was more frequent among normotensive individuals (8.4% vs. 1.0%, *P* < 0.001).^36^

In a study of 141 patients with ADPKD, 4% exhibited MVP, whereas 63% had mitral valve regurgitation, predominantly mild (88/89 cases).^28^ Tricuspid valve regurgitation was observed in 62% of patients, mostly mild (87/88 cases). In contrast, only 2% of the control group had mild tricuspid regurgitation, and no other valve defects were noted.^28^ These findings suggest a high prevalence of mild mitral and tricuspid regurgitation in ADPKD.^28^

A study comparing 271 patients with ADPKD, 271 patients with diabetic nephropathy, and 271 nondiabetic patients without ADPKD concluded that transplant recipients with ADPKD exhibit the most favorable pretransplant cardiac profile, as assessed by echocardiographic data within 2 years before kidney transplantation and the occurrence of major adverse cardiovascular events after transplantation.^5^ This is reflected in higher patient survival from major adverse cardiovascular events. However, these patients also experience a decline in valvular function and a progressive increase in Valsalva's sinus diameter, highlighting the pathogenesis as more than merely the progression of CKD, and emphasizing its association with underlying mutation-related mechanisms.^5^

Based on the KDIGO 2025 ADPKD guidelines, the recommendation for screening structural and valvular heart disease with echocardiography should be primarily guided by the presence of clinical symptoms rather than routine screening in asymptomatic patients to ensure appropriate resource utilization while enabling timely diagnosis and management in those at higher risk.^3^

### Aortic Root Dilation, Dissection, and Aneurysms

ADPKD is associated with various vascular manifestations, including aneurysms and dissections, primarily affecting the intracranial arteries and the aorta, cervicocephalic, vertebral, and coronary arteries.^42^ Oxidative stress and inflammation are prominent in patients with ADPKD and may play a role in the development of vascular dysfunction and hypertension, which is an important risk factor for aortic lesions.^43^ A study investigating the differences in the risk for an arteriovenous fistula or graft dysfunction in hemodialysis with 557 patients with ADPKD and 1671 without ADPKD demonstrated that individuals with early-stage ADPKD exhibited increased arterial stiffness and endothelial dysfunction compared with healthy controls. Importantly, administering ascorbic acid, an antioxidant, improved endothelial function in patients with ADPKD, suggesting that oxidative stress contributes to vascular dysfunction in this population. In addition, elevated expression of the inflammatory marker, NF-κB was observed in endothelial cells from patients with ADPKD, indicating increased vascular inflammation.^43^

Conversely, PCs expressed in vascular smooth muscle and endothelial cells interact with hereditary pathological pathways, which may play a significant role in the early development of vascular remodeling and aneurysms in ADPKD.^44^ Moreover, the location of the *PKD1* mutation is a significant prognostic indicator in assessing the risk of aneurysm development, with mutations in the 5′ region more frequently linked to vascular abnormalities.^43^

In a study of 2076 patients with ADPKD and 20,760 matched controls, the ADPKD group had a significantly higher frequency of atherosclerotic risk factors (i.e., hypertension, dyslipidemia, gout, and CKD) and comorbidities, except for diabetes. The incidence rate ratio of aortic aneurysm and dissection was 9.11, with a significantly higher frequency (5.5-fold higher) in the ADPKD group (0.92% vs. 0.11%, *P* < 0.0001).^44^

In another study comparing 61 patients with ADPKD with matched controls, the former were found to have a significantly higher prevalence of aortic aneurysms. Aortic dilatation (> 40 mm) was observed in 30% of patients with ADPKD versus 8% in controls. Aortic aneurysm was present in 44% of patients with ADPKD compared with 15% of controls (*P* < 0.001). Multivariate analysis identified ADPKD, lower body weight, and younger age as significant factors associated with aortic aneurysm development, with ADPKD being the strongest predictor.^42^

In a study of 97 children and young patients with ADPKD, 15.6% had aortic dilatation at the sinus of Valsalva (SoV), 17% at the sinotubular junction, 11.1% at the ascending aorta, and 5.2% at the aortic valve annulus, while none of the controls showed dilatation at these locations. Aortic dilatation was strongly associated with cyst burden, particularly at the aortic valve annulus (β = 0.42, p < 0.001) and sinus of Valsalva (β = 0.39, *P* < 0.001).^45^

### ICAs

Patients with ADPKD are at increased risk for developing ICAs, likely due to impaired vascular integrity and reduced arterial elasticity. These aneurysms typically arise as focal dilations at major arterial bifurcation points within the cerebral circulation.^46^ A previous investigation examining germline mutations in families with ADPKD and a history of ICAs identified a significant correlation between mutations located toward the 5′ end of the *PKD1* gene and an increased risk of ICA development.^47^ In this study, DNA samples from patients with ADPKD and vascular complications were systematically screened for mutations across the entire *PKD1* and *PKD2* genes. Mutations were characterized in 58 ADPKD families with vascular manifestations; 51 (88%) carried *PKD1* mutations, and 7 (12%) carried *PKD2* mutations. Notably, the median position of the *PKD1* mutation was significantly more 5′ in the vascular cohort compared with 87 control pedigrees (amino acid position 2163 vs. 2773, *P* = 0.0034).^47^ Moreover, subsets of the vascular cohort with aneurysmal rupture, early rupture, or multiple affected family members exhibited even more 5′-located mutations (median amino acid positions: 1811, *P* = 0.0018; 1671, *P* = 0.0052; and 1587, *P* = 0.0003, respectively), further supporting a potential positional effect of *PKD1* mutations on vascular risk in ADPKD.^47^ Patients with ADPKD face a markedly elevated risk, approximately 6.9 times higher, of developing ICAs compared with the general population.^3^ However, the detection of these aneurysms is often influenced by which individuals are selected for screening, leading to potential underestimation or overrepresentation in reported prevalence rates.^3^^,^^48^ The reported prevalence rates of ICAs in ADPKD range from 8% to 12%, with the risk increasing up to 22% in those with a familial history of ICAs or subarachnoid hemorrhage (SAH).^48^ Approximately 15% to 25% of patients with ADPKD who are diagnosed with an ICA are found to have multiple aneurysms.^3^

A large cohort study screened 926 patients from an ADPKD registry who underwent cerebral angiography to investigate the prevalence and risk factors of ICAs. Patients were categorized into 3 groups based on aneurysm rupture risk: aneurysm-negative, low-risk aneurysm, and high-risk aneurysm. ICAs were detected in 16.0% of patients, with 1.2% having a history of aneurysmal SAH. Among those with aneurysms, 15.5% were classified as low-risk and 84.5% as high-risk. Multivariate analysis identified age (odds ratio [OR]: 1.03, 95% CI: 1.01–1.05, *P* = 0.004), female sex (OR: 3.13, 95% CI: 1.94–5.06, *P* < 0.001), dolichoectasia (OR: 8.57, 95% CI: 1.53–48.17, *P* = 0.015), and mitral inflow deceleration time (OR: 1.01, 95% CI: 1.00–1.01, *P* = 0.046) as independent factors positively associated with high-risk aneurysms. Conversely, hypercholesterolemia was inversely related to high-risk aneurysm formation (OR: 0.46, 95% CI: 0.29–0.72, *P* = 0.001).^49^

Another large cohort study reviewed the records of 3010 patients with ADPKD who were evaluated between 1989 and 2017 to assess the prevalence and progression of ICAs. Among 812 individuals who underwent presymptomatic magnetic resonance angiography screening, 94 ICAs were identified in 75 patients, yielding a prevalence of 9%. Demographic variables, including age, sex, race, and genotype, did not differ significantly between patients with and without aneurysms. However, hypertension, smoking history, and a family history of SAH were more common in those with aneurysms, with 29% of aneurysm-positive patients reporting a family history of SAH compared with 11% in the aneurysm-negative group (*P* < 0.001). The majority of aneurysms were small (median diameter: 4 mm) and located in the anterior circulation (85%). Notably, among 737 patients with negative initial magnetic resonance angiography results, only 2 experienced aneurysmal rupture over 4783 clinical follow-up years, corresponding to a rupture rate of 0.04/ 100 patient-yrs (95% CI: 0–0.10).^50^

The KDIGO guidelines recommend ICA screening for patients with a personal history of SAH or a positive family history of ICA, SAH, or unexplained sudden death, provided they are eligible for treatment and have a reasonable life expectancy (grade 1D).^3^ Targeted screening for patients considered high risk or classified as grade 1D between the ages of 20 and 70 with a good life expectancy involves the use of brain magnetic resonance angiography or computed tomography angiography and is strongly recommended.^48^ Other risk factors that warrant screening include smoking, hypertension, and certain ancestries (e.g., Finnish or Japanese).^48^ For patients without these high-risk features, screening may still be considered based on age and individual clinical context. If an ICA is detected, further management is guided by aneurysm characteristics such as size (particularly 5–7 mm) and the presence of symptomatic clinical features. Referral to a multidisciplinary team involving neurology, neurosurgery, and neuroradiology is required. Repeat imaging is advised if neurological symptoms develop or if changes in ICA morphology are observed.^48^ In addition, KDIGO suggests that screening should be considered in individuals with *de novo* ADPKD, an unknown family history, a limited number of affected relatives, or a personal or familial history of extracerebral vascular abnormalities. Furthermore, it recommends discussing screening in the context of pretransplant evaluations or before a major elective surgery.^3^

### PCE

PCEs are an underrecognized cardiac complication of ADPKD that might emerge before the decline in kidney function.^51^ The prevailing hypothesis attributes PCEs in ADPKD to mutations in the *PKD1* gene. These mutations may lead to defective extracellular matrix components and altered expression of matrix-degrading enzymes and their inhibitors, resulting in increased compliance and impaired recoil of the parietal pericardium. This structural laxity facilitates the accumulation of pericardial fluid, contributing to the development of PCE in affected patients with ADPKD.^52^

Liu *et al.*^51^ evaluated 117 patients with ADPKD and matched controls. The prevalence of PCE > 5 mm was 21% (24 of 117), and it was present in 17% of patients (8/46) with ADPKD who had both magnetic resonance imaging and echocardiography. Results showed significant correlations between PCE and gender (*P* = 0.03), right pleural effusion (*P* < 0.0001), and negative correlation with age. Right pleural effusion and female gender were associated with a higher rate of PCEs. Magnetic resonance imaging detected asymmetrically distributed PCEs in 5 cases not reported on echocardiography. Genotype analysis of 75 patients with ADPKD revealed no significant correlation between genotype and PCEs. Importantly, no patient required pericardiocentesis or any intervention for PCE.

Another study reported 8.2% (17 patients) PCEs in 286 patients with ADPKD, with an overall prevalence of 6.3%, with females affected predominantly. This study also showed that PCE was not correlated with signs of disease progression and ADPKD genotype.^53^

### Coronary Artery Disease

Coronary artery disease (CAD) is a significant concern for patients with ADPKD, because cardiovascular complications are among the leading causes of morbidity and mortality in this population.^54^ The relationship between ADPKD and CAD is multifaceted, involving shared risk factors such as hypertension, dyslipidemia, and LVH, which are prevalent in patients with ADPKD.^54^ The presence of LVH is a significant risk factor for the development of CAD, because it is associated with increased myocardial oxygen demand and can lead to ischemic heart disease. Studies have shown that CAD is responsible for a substantial proportion of deaths in patients with ADPKD.^41^ Patients with ADPKD have been reported to develop ischemic heart disease before the onset of kidney failure. Moreover, there are documented reports of coronary aneurysms and dissections.^55^ This highlights the importance of regular cardiovascular screening and management in patients with ADPKD. Preventive strategies, including tight management of hypertension and dyslipidemia, are crucial to mitigate the risk of CAD.^55^ In addition to classical atherosclerotic CAD, there are reports of coronary artery aneurysms and spontaneous coronary artery dissections in this population. A review of reported spontaneous coronary artery dissection cases in ADPKD indicated a predominance in females with a median age of 41 years, frequently involving the left anterior descending artery; however, the genetic status of these patients was not established.^55^ This observation may suggest that beyond traditional atherosclerotic disease, structural vascular abnormalities, including coronary dissection, may represent an underrecognized but clinically relevant cardiovascular manifestation of ADPKD. Therefore, differentiating the various causes of CAD, including atherosclerosis versus vascular fragility or dissection, is important for a more nuanced understanding of cardiovascular risk in ADPKD and should be emphasized in future investigations.

### Heart Failure

The activation of the RAAS, resulting in hypertension, significantly contributes to the progression of heart failure (HF).^56^ Diastolic HF, also referred to as HF with preserved ejection fraction, represents the most prevalent form of HF in rare kidney diseases, progressing to CKD and is an independent predictor of mortality within this population.^23^ The primary physiological hallmark of diastolic HF is an elevation in left ventricular filling pressure.^23^ However, the direct prevalence of HF in ADPKD has yet to be specifically reported.^56^ In contrast, while comprehensive studies specifically examining the prevalence of HF in ADPKD are limited, studies that have reported data on left ventricular ejection fraction (LVEF) offer valuable insights into cardiac function within this population.^56^

A study assessed 667 patients with ADPKD with available echocardiographic data identified 39 cases (5.8%) of idiopathic dilated cardiomyopathy and 17 cases (2.5%) of hypertrophic obstructive cardiomyopathy. In addition, 2 patients (0.3%) were diagnosed with left ventricular noncompaction, both with *PKD1* mutations.^6^ The study did not specifically investigate diastolic dysfunction. The mean age at diagnosis of idiopathic dilated cardiomyopathy was 53.3 ± 12.1 years, with 57% of affected individuals being male. The median LVEF at initial diagnosis was 25%. Genetic analysis revealed *PKD1* truncating mutations in 31.6% of idiopathic dilated cardiomyopathy cases, *PKD1* nontruncating mutations in 15.8%, and *PKD2* mutations in 36.8%.^6^

The German AD(H)PKD registry, which evaluated 141 patients with ADPKD, reported a higher mean LVEF of 63% compared with 60% in the control group, which is consistent with the presence of a functionally hypertrophied myocardium. Given the relatively young mean age of the ADPKD cohort (44 years), it remains uncertain whether LVEF will decline over time, potentially progressing toward HF with reduced ejection fraction. Notably, the study excluded 3 individuals with LVEF values < 30%, which may have influenced the overall assessment of systolic function in this population.^22^

In Table 1,^5^^,^^35^^,^^38^^,^^39^^,^^42^^,^^44^^,^^45^^,^^53^^,^^54^^,^^57^, ^58^, ^59^, ^60^, ^61^, ^62^ we summarize key cardiovascular manifestations associated with ADPKD, including their reported prevalence in affected individuals; and in Table 2,^3^ we provide key points and current screening recommendations outlined in the KDIGO 2025 ADPKD guidelines.^3^

**Table 1:** Prevalence of cardiac complications in patients with ADPKD that has been reported by previous studies

Cardiac complications	Population	Prevalence range	Study
Hypertension	281 patients with ADPKD (*PKD1* mutations) Definition: systolic BP > 150 mm Hg, or diastolic BP > 90 mm Hg at the study visit.	70%	Gabow *et al.*^57^
	16 patients with ADPKD, age range: 20–50 yrs with mean age in hypertensive group: 38.6 ± 2.2 yrs	50%–75%	Bell *et al.*^58^
Left ventricular hypertrophy	126 patients with ADPKD Age > 18 yrs, median age: 46 yrs, eGFR > 15	21.4%	Chen *et al.*^35^
	31 kidney transplant recipients with ADPKD, 41 non-ADPKD controls, 14 women in ADPKD group and 13 in controls	74%	Jankowska *et al.*^59^
	271 kidney transplant recipients with ADPKD, 271 kidney transplant recipients with diabetes, 271 kidney transplant recipients without ADPKD and diabetes, Age > 18 yrs, mean age (SD): 57.2 ± 8.8 yrs, 59.4% male	39.4%	Chedid *et al.*^5^
Aortic root dilation	97 patients with ADPKD, 19 controls without ADPKD, Age < 18 yrs, Median age (IQR): 9.3 (6.1–13.6) yrs, 41% male, 65% White	5.2% to 17%	Savis *et al.*^45^
Aortic dissection	2076 patients with ADPKD, 2076 non-ADPKD, Age > 18 yrs, Median age (IQR): 47 (38-56) yrs, 51.64% female, 48.36% male	58%	Sung *et al.*^44^
Aortic aneurysm	61 patients with ADPKD, 61 controls mean age (SD) in ADPKD group: 56 ± 12 yrs, 46% female, 54% male	44%	Bouleti *et al.*^42^^,^^60^
Abdominal aortic aneurysm	63.2% female, 34.6% male, 2.2% not reported	0.8%	Helal *et al.*^54^
Valvular abnormalities	63.2% female, 34.6% male, 2.2% not reported	14.4%	Helal *et al.*^54^
Arrhythmias	419 patients with ADPKD, mean age: 53.2 ± 13.7 yrs, 63.2% female, 34.6% male, 2.2% not reported	25.9%	Helal *et al.*^54^
Mitral valve prolapse	102 patients with ADPKD, Age < 18 yrs, mean age (SD): 10.3 ± 5.3 yrs,	25%	Savis *et al.*^38^
	109 patients with ADPKD with *PKD1* mutation, 73 unaffected family members, 73 controls, age in ADPKD group: 44 ± 1 (16–79) yrs	26%	Lumiaho *et al.*^39^
Mitral regurgitation	109 patients with ADPKD with *PKD1* mutation, 73 unaffected family members, 73 controls, age in ADPKD group: 44 ± 1 (16–79) yrs	3% to 13%	Lumiaho *et al.*^39^
Coronary atherosclerosis	132 patients with ADPKD, 498 controls, 1121 patients with CKD from other etiologies, Age: 53.60 ± 11.09, 50.8% male, 43.2% CKD stage 3, 56.8% CKD stages 4–5	53.8%	Gorriz *et al.*^61^
Myocardial infarction	2062 patients with ADPKD, 20,620 non-ADPKD, age > 18 yrs, median age (IQR) in ADPKD group: 47 (38–56), 51.5% female, 48.5% male	2.9%	Sung *et al.*^62^
	419 patients with ADPKD, mean age: 53.2 ± 13.7 yrs, 63.2% female, 34.6% male, 2.2% not reported	6%	Helal *et al.*^54^
Pericardial Effusion (PCE)	208 patients with ADPKD, Patients without PCE % (*n*): 91.8 (191), Patients with PCE % (*n*): 8.2 (17), 19 yrs < age < 77 yrs	8.2%	Jost *et al.*^53^
Heart failure	419 patients with ADPKD, mean age: 53.2 ± 13.7 yrs, 63.2% female, 34.6% male, 2.2% not reported	9.5%	Helal *et al.*^54^
	2062 patients with ADPKD, 20,620 non-ADPKD, Age > 18 yrs, median age (IQR) in ADPKD group: 47 (38-56) yrs, 51.5% female, 48.5% male	10.5%	Sung *et al.*^62^

ADPKD, autosomal dominant polycystic kidney disease; CKD, chronic kidney disease; IQR, interquartile range.

**Table 2:** Cardiac manifestations of ADPKD outlining the proportion of patients with ADPKD for each category and guidance for screening from KDIGO 2025 ADPKD Guidelines.^3^

Cardiovascular manifestation	Estimation of the % of patients affected by ADPKD	Key notes	Guidance for imaging
Mitral valve prolapse (MVP) and regurgitation	MVP: 3%–26%	• Usually asymptomatic • MVP was formerly reported to be present in 20%–30% of people with ADPKD. Still, more recent studies using the current definition of MVP reported a prevalence of 1% in pediatric and 3.4% in adult cohorts, similar to the general population.	No systematic screening
Pericardial effusion	∼ 20%	• Usually asymptomatic, incidental diagnosis	No systematic screening
Cardiomyopathy	Rare	• Hypertrophic cardiomyopathy: 2.5%^a^ • Dilated cardiomyopathy: 5.8%^a^ • Left ventricular noncompaction: 0.3%	No systematic screening
Congenital heart malformation	Very rare	• Very rare case series and case reports • Left-to-right shunt, obstructive cardiomyopathies (aortic coarctation, congenital pulmonic stenosis), and other complex malformations have been reported.	No systematic screening
Situs inversus and large vessels transposition	Rare case reports	• Laterality defects including dextrocardia and situs inversus totalis have been reported in a small number of people with ADPKD, mostly *PKD2* (genetic testing was not performed in all reported cases	No systematic screening
Thoracic aortic aneurysm (TAA)	∼ 1.5%	• In patients eligible for screening (e.g., first-degree relatives of a person with a diagnosis of TAA), a contrast-enhanced CT scan or MRA can be performed. • Transthoracic echocardiography is a standard approach to identifying and monitoring aortic root dilatation.	No systematic screening. To be considered in case of positive familial history
Thoracic aortic dissection	Very rare case reports	• Acute chest/upper back/abdominal pain is present in > 90% of the cases.	Only if symptoms are present
Coronary artery dissection	Very rare case reports	• Patients generally present with symptoms and signs characteristic of acute myocardial infarction. • Usually more frequent in young women	Only if symptoms are present
Carotid and vertebral artery dissection	Very rare case reports	• Often, it results in ischemic stroke or transient ischemic attack associated with neck pain or headaches. • Occasional Horner syndrome in case of carotid dissections	Only if symptoms are present

ADPKD, autosomal dominant polycystic disease; CT, computed tomography; KDIGO, Kidney Disease Improving Global Outcomes; MRA, magnetic resonance angiography.

a Estimates taken from a single study and should be considered with caution.

### Potential Cardiovascular Benefits of Vasopressin Receptor Antagonism, Sodium-Glucose Cotransporter Inhibitors, and Mineralocorticoid Receptor Antagonists in ADPKD

Vasopressin acts on 3 receptors identified across various tissues, with V2R and V1aR being particularly relevant to HF and polycystic kidney disease.^63^ V1aRs are present in vascular smooth muscle cells and cardiac myocytes, where they contribute to pressor effects and myocyte hypertrophy. They are also found in the collecting duct principal cells, where they mediate diuretic and natriuretic effects.^63^ Aquaretics, including dual V1aR/V2R and selective V2R antagonists, act on V2R. These agents increase free water clearance and correct hyponatremia and have gained attention for treating congestive HF and are the only medical treatments approved for slowing ADPKD progression.^4^ Tolvaptan, as a V2R antagonist, reduces right atrial and pulmonary artery wedge pressures.^63^ According to a systematic review, tolvaptan was associated with short-term improvement in congestive symptoms, correction of hyponatremia, and a reduced incidence of worsening kidney function in patients with acute HF, with no significant benefits observed in long-term symptoms.^64^ Therefore, although it has not demonstrated a survival benefit, it may be considered an effective therapeutic option in patients with ADPKD with hyponatremia and HF, because of the advantages conferred by its unique mechanism of action.^4^

Whether tolvaptan therapy could play a role in delaying cardiovascular disease in patients with ADPKD is yet to be seen. A pilot study showed that tolvaptan treatment reduced markers of early atherosclerosis and endothelial dysfunction, such as carotid intima-media thickness and epicardial adipose tissue thickness, while improving flow-mediated dilation and reducing inflammation (C-reactive protein levels).^65^ Another study evaluating long-term kidney and cardiovascular effects of tolvaptan therapy reported a potential role in preventing arrhythmias by inhibiting an increase in QTc interval and heart rate after a 3-year follow-up, in addition to slowing kidney progression in ADPKD.^66^ However, more studies are needed to confirm these cardiovascular benefits and to better understand the mechanisms underlying these effects in patients with ADPKD.

Alternatively, sodium-glucose cotransporter-2 (SGLT2) inhibitors may offer multiple potential benefits in ADPKD by targeting various pathophysiological pathways. These agents enhance natriuresis, which can reduce intraglomerular pressure through tubuloglomerular feedback, leading to a decrease in the urinary albumin-to-creatinine ratio and promoting better BP control. Increased glycosuria induced by SGLT2 inhibition contributes to glycemic regulation and induces mild ketosis, potentially leading to moderate weight loss, both of which are advantageous in metabolic management and may attenuate cystogenesis. Additional proposed mechanisms include improved mitochondrial efficiency and reduced oxidative stress and inflammation, particularly via downregulation of interleukin-6 and other proinflammatory cytokines, as well as mitigation of interstitial fibrosis.^67^ According to current KDIGO recommendations, SGLT2 inhibitors should not be used to slow eGFR decline in patients with ADPKD.^3^ However, a recent retrospective cohort study of 348 patients with ADPKD (93% male; mean age ± SD, 68 ± 11 years) demonstrated that those who initiated SGLT2 inhibitors experienced a slower rate of eGFR decline, particularly beyond the initial 3 months of therapy. After an early decrease in eGFR (−2.78 ml/min per 1.73 m^2^/ 90 d), kidney function stabilized between months 3 and 12 (−0.07 ml/min per 1.73 m^2^/90 d). In patients with concomitant type 2 diabetes, SGLT2 inhibitor use was associated with a significantly slower eGFR decline compared with dipeptidyl peptidase-4 inhibitors during the same period (difference: +1.29 ml/min per 1.73 m^2^/90 d), suggesting potential benefit in individuals where diabetes and cardiovascular comorbidities may contribute more prominently to kidney disease progression.^68^ Nonetheless, SGLT2 inhibitors are well-established as cardioprotective and renoprotective agents in both diabetic and nondiabetic populations.^69^ Given these broad therapeutic effects, patients with ADPKD may theoretically experience similar cardiovascular and kidney protection.^69^ However, the potential benefits of SGLT2 inhibitors have not been specifically evaluated in the ADPKD population, because major clinical trials in CKD without diabetes have excluded individuals with this condition.^3^ Despite this, SGLT2 inhibitors may still be appropriately prescribed to patients with ADPKD for approved cardiovascular indications, reflecting their potential efficacy in this group. Currently, human data on ADPKD are limited to observational studies. A randomized controlled trial (NCT05510115) is underway to assess the safety and efficacy of SGLT2 inhibition in this population.^3^^,^^67^ Further research is also warranted to elucidate the metabolic effects of SGLT2 inhibition in this specific population.

Mineralocorticoid receptor antagonists have demonstrated cardiovascular benefits in various populations; however, data on their use in patients with ADPKD remain limited. A randomized controlled trial evaluated the impact of spironolactone in 60 individuals with early-stage ADPKD (mean age: 34 ± 10 years; 54% women; 84% non-Hispanic White) over 24 weeks. The study found no significant improvement in vascular endothelial function or large artery stiffness after 24 weeks of treatment despite a modest reduction in systolic BP (median change: −6, interquartile range: −15 to 1] mm Hg vs. −2, interquartile range: −7 to 10 mm Hg; *P* = 0.04).^70^ No meaningful changes were observed in circulating or endothelial cell markers of oxidative stress or inflammation. Although this study did not demonstrate vascular benefits, it highlighted the safety and tolerability of mineralocorticoid receptor antagonists in this population.^70^ Emerging nonsteroidal mineralocorticoid receptor antagonists with greater selectivity and improved safety profiles may offer the potential to slow the decline in kidney function and mitigate cardiovascular risk in ADPKD. However, clinical trials evaluating these agents, specifically in ADPKD, are currently lacking, and further research is warranted to explore their long-term effects on the kidneys and cardiovascular system.

### Conclusion

The pathogenesis of cardiovascular complications is multifaceted, and early and effective BP control is crucial in preventing cardiovascular risks. Valvular heart disease in ADPKD has a bimodal age distribution with the early stages related to gene mutations. This concept has a clinical implication in close follow-up of posttransplant patients because there is a chance for the occurrence of cardiac involvement even posttransplantation. In alignment with the KDIGO 2025 ADPKD guidelines, baseline echocardiography, with repeat assessments as clinically indicated, is recommended for patients with ADPKD who present with a history of severe or uncontrolled hypertension, cardiac murmurs, signs or symptoms suggestive of cardiac dysfunction, other cardiovascular manifestations, or a family history of thoracic aortic aneurysm or nonischemic cardiomyopathy. Long-term studies are needed to explore the association between genetic mutations and cardiac findings and the role of tolvaptan and novel therapies on preventing cardiovascular disease to better understand the cardiovascular complications linked to specific genotypes, enabling earlier detection, personalized management, and improved outcomes in these genetically predisposed populations.

## Disclosure

YC is a member of the Nephronomics Scientific Advisory Board. PSG receives honoraria from Otsuka Inc and serves as the co-chair for the PKD Centers of Excellence Advisory Committee. FTC has received research funding from Otsuka Pharmaceuticals, Regulus, and Natera Inc. He serves as a member of the Board of Directors for the PKD Foundation and as a co-chair for the PKD Centers of Excellence Advisory Committee. NKD is a consultant for Regulus, Natera, and Vertex. AR has received advisory board and speakers’ bureau fees from AstraZeneca, Baxter, Bayer, GlaxoSmithKline, Sanofi, Natera, and Travere Therapeutics; has received research grants from AstraZeneca, Bayer, Palladio Biosciences, Reata Pharmaceuticals, Regulus Therapeutics, and Sanofi; and has received consulting fees from AstraZeneca, GlaxoSmithKline, Novo Nordisk, Otsuka, Sanofi, and Travere Therapeutics. SN receives honoraria from Otsuka Inc. All the other authors declared no competing interests.

## Data Availability Statement

The data underlying this article are available in the article itself.

## Author Contributions

NE and SN drafted the manuscript. YC, SC, PSG, FTC, NKD, AR, and AA reviewed and edited the manuscript. GHB contributed to the conception, review, and editing of the manuscript. SN contributed to the conception, review, and supervision of the manuscript
